# AST to ALT ratio and arterial stiffness in non-fatty liver Japanese population:a secondary analysis based on a cross-sectional study

**DOI:** 10.1186/s12944-018-0920-4

**Published:** 2018-12-03

**Authors:** Yongmei Liu, Peiling Zhao, Mingliang Cheng, Lei Yu, Zhuo Cheng, Linda Fan, Chi Chen

**Affiliations:** 1 Department of Infectious Diseases, The Affiliated Hospital of Guizhou Medical University, 28# Guiyi Road, Guiyang, 550004 Guizhou China; 2 Prenatal Diagnosis Center, Hospital Affiliated to Guizhou Medical University, 2# Beijing Road, Guiyang, 550004 China; 3 Department of Clinical Medicine, Peking University Health Science Center, Peking University, Beijing, 100191 China; 4 Department of Immunology and Microbiology, Guiyang College of Traditional Chinese Medicine, 84# ShiDong Road, Guiyang, 550001 Guizhou China

**Keywords:** Aspartate aminotransferase, Alanine aminotransferase, Brachial-ankle pulse wave velocity, Association

## Abstract

**Background:**

Previous studies have revealed that triglyceride to high-density lipoprotein cholesterol (HDL-C) ratio (henceforth TG/HDL-C) is one of the major risk factors for cardiovascular disease, insulin resistance and metabolism syndrome. However, there are fewer investigations of the correlations between the aspartate aminotransferase (AST) to alanine aminotransferase (ALT) ratio and brachial ankle pulse wave velocity (baPWV). This study was undertaken to investigate the relationship between the AST to ALT ratio and brachial-ankle pulse wave velocity (baPWV) in a Japanese population.

**Methods:**

The present study was a cross-sectional study. A total of 646 Japanese men and women without fatty liver, aged 24—84 years old, received a health medical check-up programme including the results from baPWV inspection and various standardized questionnaires in a health examination centre in Japan. Main outcome measures included AST/ALT ratio, baPWV, fatty liver and postmenopausal status. Abdominal ultrasonography was used to diagnose fatty liver. A postmenopausal state was defined as beginning 1 year following the cessation of menses.

**Results:**

After adjusting for potential confounders (age, sex, BMI, SBP, DBP, AST, ALT, GGT, uric acid, fasting glucose, TC, LDL, eGFR, smoking and exercise statuses, fatty liver, alcohol consumption and ABI), a non-linear relationship was detected between AST/ALT and baPWV, which had an inflection point of 5.6. The effect sizes and the confidence intervals on the left and right sides of the inflection point were 12.7 (1.9 to 23.5) and − 16.7 (− 36.8 to 3.3), respectively. Subgroup analysis in participants with excessive alcohol consumption (more than 280 g/week) showed that AST/ALT had a negative correlation with baPWV (β = − 30.7, 95%CI (− 53.1, − 8.4)), and the *P* value for the interaction was less than 0.05.

**Conclusion:**

The relationship between AST/ALT and baPWV is non-linear. AST/ALT was positively correlated with baPWV when AST/ALT was less than 5.6. In addition, the trend was the opposite in subjects who consumed excessive amounts of alcohol.

## Background

Brachial-ankle pulse wave velocity (baPWV) is employed to evaluate arterial stiffness [1]. To date, a number of studies have confirmed that baPWV is an independent risk factor for cardiovascular events, and it is used in clinical settings for early evaluation of the functions and structural changes of vascular walls [2–4]. Although western countries have not fully accepted baPWV, an increasing number of publications on this research methodology have come from these countries since 2009. Two well-known examples with respect to baPWV utilities are the Atherosclerosis Risk in Communities (ARIC) study and the Bogalusa Heart Study [5]. The two large-scale studies in the U.S. have used baPWV as an indicator to assess arterial stiffness.

There is a consensus that liver function enzymes, such as aspartate aminotransferase (AST) and alanine aminotransferase (ALT), are biomarkers that reflect disease severity in a number of chronic liver diseases. More recently, Stephen F Weng, et al. [6] revealed that elevated AST/ALT ratios (henceforth AST/ALT) are independently associated with increased risk of developing cardiovascular disease (CVD) within 10 years in men but not in women. Given that AST/ALT and baPWV are both correlate with CVD, we hypothesize that AST/ALT may be associated with baPWV.

In this study, we performed a secondary data analysis based on previously published data [7]. In that paper, the author investigated the correlation between γ-glutamyltranspeptidase and baPWV. On secondary analysis, AST/ALT was used as an independent variable, and outcome variables and other covariates are consistent with those in the original analysis.

## Methods

### Data source

Data were obtained from the ‘DATADRYAD’ database (www. Datadryad.org), a website that permits users to freely download raw data. Authors of the original study have waived all copyright and related ownership of these data. Therefore, we could use these data for secondary analysis without infringing on the authors’ rights. When we used these data, we cited the Dryad data package (Dryad data package: Fukuda T, Hamaguchi M, Kojima T, Ohshima Y, Ohbora A, Kato T, Nakamura N, Fukui M (2014) Data from: Association between serum γ-glutamyltranspeptidase and atherosclerosis: a population-based cross-sectional study. Dryad Digital Repository. 10.5061/dryad.m484p). Variables included in the database file were as follows: age, diastolic blood pressure (DBP), body mass index (BMI), alanine aminotransferase (ALT), systolic blood pressure (SBP), aspartate transaminase (AST), γ-glutamyltranspeptidase (GGT), fasting glucose, uric acid, total cholesterol (TC), low density lipoprotein (LDL), baPWV, estimated glomerular filtration rate (eGFR), sex, smoking status, exercise status, fatty liver disease, menopausal status, high-density lipoprotein cholesterol (HDL-C), alcohol consumption, ankle-brachial index (ABI) and triglyceride (TG).

### Study population

This is a cross-sectional study conducted by Takuya Fukuda et al [7] at the Medical Health Checkup Center of Murakami Memorial Hospital, Gifu City, Japan, from March 2004 to December 2012. Participants received a medical health check-up programme including pulse wave velocity and abdominal ultrasonography. A total of 1445 participants were recruited and selected according to standard exclusion criteria, as follows: (1) the participants received hormone replacement therapy; (2) the participants took oral contraceptives; (3) the participants had positive hepatitis B and hepatitis C viral antigens; (4) the participants were of gestational age; (5) the participants had an ankle-brachial index (ABI) less than 0.95; and (6) the participants were diagnosed with fatty liver. Researchers obtained information (values) for baPWV, AST/ALT and other covariants at baseline. Because the study was a second analysis of the existing data that were anonymous, there is no need to obtain informed consent from the participants.

### Measurement of baPWV, AST/ALT and other covariants

Takuya Fukuda et al. [7] completed the entire study. To understand the entire research process more clearly, we have outlined the steps of the study here.

ABI and baPWV were measured using an automatic waveform analyser (Colin Medical Technology, Komaki, Japan). The subjects were in the supine position and rested in a quiet and suitably heated room for 5 min. We then placed ECG electrodes and heart sound microphone on both wrists and the left edge of the sternal border, respectively. Cuffs connected with plethysmographic sensors and oscillometric pressure sensors were wrapped around the arms and ankles. From this, Takuya Fukuda et al. calculated the path lengths from the suprasternal notch to the brachium (Lb) and from the suprasternal notch to the ankle (La) and then automatically obtained the delay time from the ascending point of the brachial waveform to the ascending point of each ankle waveform (DTba). Finally, they calculated baPWV by the formula (La-Lb)/DTba. The measurement and assessment of AST/ALT and other covariants were described in detail in the original study.

Takuya Fukuda et al. diagnosed fatty liver by abdominal ultrasonography (Aloka SSD-650CL (Aloka Co, Ltd., Tokyo, Japan). One gastroenterologist diagnosed fatty liver by ultrasonographic images stored in a computer without reference to other individual data of any of the participants. Of the four known criteria (hepatorenal echo contrast, liver brightness, deep attenuation and vascular blurring), the participants were required to have hepatorenal contrast and liver brightness to be given a diagnosis of fatty liver [8].

### Statistical analysis

The total procedure of statistical analysis was divided into five steps. First, we analysed baseline characteristics of participants according to following principles (we grouped AST/ALT in quartiles): (1) continuous variables were expressed as the means ± standard deviations (normal distribution) or medians (quartiles) (skewed distribution), and categorical variables were expressed as a frequency or percentages; and (2) the one-way ANOVA (normal distribution), Kruskal-Wallis H (skewed distribution) test and chi-square test (categorical variables) were used to determine any significant differences between the means and proportions of the groups. It was noted that the AST/ALT value was too small; therefore, we expanded it 10 times and labelled per 0.1 change (henceforth AST/ALT per 0.1 change). Second, we used a univariate linear regression model to evaluate the associations between AST/ALT and baPWV. Third, according to the recommendation of the STROBE statement [8], we simultaneously showed the results from unadjusted, minimally adjusted analyses and those from fully adjusted analyses. The covariances, when added to this model, changed the matched odds ratio by at least 10% and were adjusted [9]. Fourth, generalized additive models (GAM) were used to identify non-linear relationships because AST/ALT was a continuous variable. If a non-linear correlation was observed, a two-piecewise linear regression model was performed to calculate the threshold effect of the AST/ALT on baPWV in terms of the smoothing plot. When the ratio between baPWV and AST/ALT appeared obvious in a smoothed curve, the recursive method automatically calculates the inflection point, where the maximum model likelihood will be used [10]. Fifth, subgroup analyses were performed using stratified linear regression models. The modifications and interactions of subgroups were inspected by likelihood ration tests. All of the analyses were performed with the statistical software package R (http://www.R-project.org, The R Foundation) and EmpowerStats (http://www.empowerstats.com, X&Y Solutions, Inc., Boston, MA). *P* values less than 0.05 (two-sided) were considered statistically significant.

## Results

### The selection of participants

**Fig. 1:**
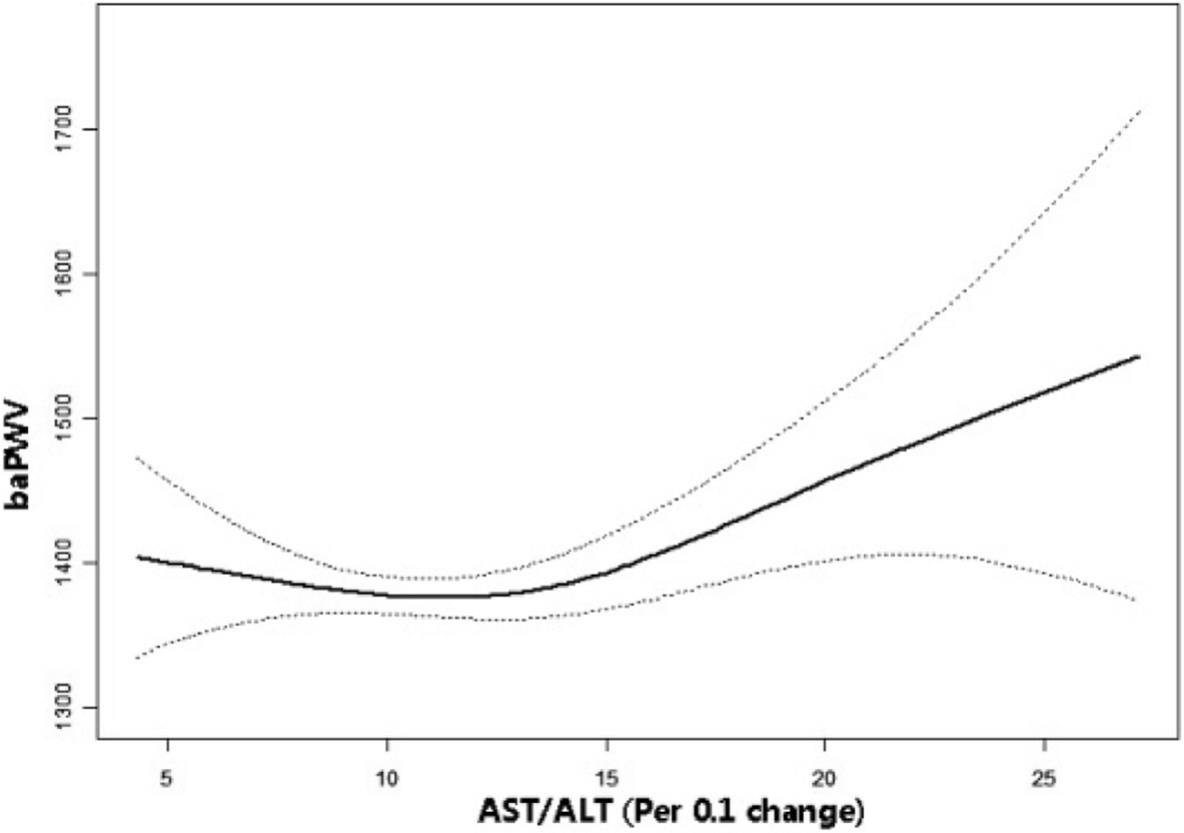
The non-linear relationship between AST/ALT and baPWV. The relationship between AST/ALT and baPWV. A nonlinear relationship between them was detected after adjusting for age, sex, BMI, SBP, DBP, AST, ALT, GGT, uric acid, fasting glucose, TC, LDL, eGFR, smoking and exercise statuses, fatty liver, alcohol consumption and ABI

Of the 1445 participants, 799 were excluded from this study. Of the 799 excluded subjects, 433 received medication, 1 took an oral contraceptive, 66 received hormone replacement therapy, 26 were positive for hepatitis B and hepatitis C antigens, 1 was of gestational age, and 6 had ABIs less than 0.96. A total of 266 were diagnosed with fatty liver, leaving 646 subjects for data analysis (Fig. 1).

### Baseline characteristics of participants

The average age of the participants was 51.5 ± 9.6 years old, and approximately 57.6% of them were male. Baseline characteristics are listed in Table 1. There was no statistically significant difference in SBP, TC, LDL, e-GFR, baPWV or ABI among the different AST/ALT groups. Compared with the low level (Q4) AST/ALT group, patients had a significantly higher BMI, DBP, GGT, uric acid, fasting glucose, TC, LDL, eGFR, baPWV and ABI in the other three groups (Q1-Q3). In the population with lower AST/ALT (Q1, Q2), men accounted for the majority, and more subjects were not current smokers compared with those in the Q3 and Q4 groups.

**Table 1:** Baseline Characteristics of participants

AST/ALT	Q1	Q2	Q3	Q4	*P*-value	*P*-value*
N	89	138	207	212		
AGE (years, mean ± sd)	49.0 ± 9.7	50.7 ± 8.6	51.7 ± 9.5	52.7 ± 10.0	0.017	0.015
BMI (kg/m2, mean ± sd)	23.1 ± 2.2	22.5 ± 2.5	22.0 ± 2.4	21.4 ± 2.3	< 0.001	< 0.001
SBP (mmHg, mean ± sd)	120.7 ± 16.3	118.8 ± 13.0	116.8 ± 15.3	116.0 ± 13.8	0.040	0.071
DBP (mmHg, mean ± sd)	76.5 ± 10.0	76.1 ± 9.3	73.6 ± 9.9	72.7 ± 9.7	0.001	< 0.001
GGT (IU/L, mean ± sd)	21.0(17.0–40.0)	21.5(15.0–33.0)	16.0(12.0–20.0)	14.0 (11.0–18.0)	< 0.001	< 0.001
Uric acid (mg/dl, mean ± sd)	5.2 ± 1.2	5.5 ± 1.4	4.7 ± 1.2	4.8 ± 1.2	< 0.001	< 0.001
Fasting glucose (mg/dL, mean ± sd)	98.2 ± 9.4	96.8 ± 9.6	95.6 ± 9.0	92.4 ± 7.5	< 0.001	< 0.001
TC (mg/dL, mean ± sd)	206.4 ± 32.5	207.1 ± 35.7	206.1 ± 35.2	207.8 ± 36.2	0.970	0.935
LDL (mg/dL, mean ± sd)	127.1 ± 29.9	125.3 ± 31.2	123.4 ± 30.2	122.6 ± 32.1	0.646	0.594
eGFR(mL/min/1.73 m^2^, mean ± sd)	71.0 ± 12.2	70.1 ± 11.3	71.9 ± 11.7	70.5 ± 12.9	0.498	0.434
baPWV (cm/s, mean ± sd)	1403.4 ± 270.1	1383.0 ± 216.9	1380.7 ± 225.9	1393.5 ± 250.3	0.866	0.844
SEX (n,%)					< 0.001	–
male	77 (86.5%)	105 (76.1%)	103 (49.8%)	87 (41.0%)		
female	12 (13.5%)	33 (23.9%)	104 (50.2%)	125 (59.0%)		
Current smokig (n,%)					< 0.001	–
none	62 (69.7%)	98 (71.0%)	170 (82.1%)	187 (88.2%)		
Current	27 (30.3%)	40 (29.0%)	37 (17.9%)	25 (11.8%)		
Ex-Smoking (n,%)					< 0.001	–
no	32 (36.0%)	60 (43.5%)	125 (60.4%)	129 (60.8%)		
yes	57 (64.0%)	78 (56.5%)	82 (39.6%)	83 (39.2%)		
Regular Exercise (> 1 week) (n,%)					0.039	–
No	78 (87.6%)	104 (77.0%)	155 (77.1%)	151 (72.2%)		
Yes	11 (12.4%)	31 (23.0%)	46 (22.9%)	58 (27.8%)		
Post-Menopausal (n,%)					0.692	–
no	7 (58.3%)	17 (51.5%)	45 (43.3%)	57 (45.6%)		
yes	5 (41.7%)	16 (48.5%)	59 (56.7%)	68 (54.4%)		
Alcohol consumption (n,%)					0.043	–
< 40 (g/week)	59 (66.3%)	72 (52.9%)	148 (71.8%)	140 (66.7%)		
≤ 40–140	16 (18.0%)	29 (21.3%)	31 (15.0%)	31 (14.8%)		
> 140–280	4 (4.5%)	18 (13.2%)	15 (7.3%)	20 (9.5%)		
> 280	10 (11.2%)	17 (12.5%)	12 (5.8%)	19 (9.0%)		
ABI	1.2 ± 0.1	1.2 ± 0.1	1.2 ± 0.1	1.3 ± 0.8	0.055	< 0.001

*ALT* alanine aminotransferase, *AST* aspartate transaminase, *baPWV* brachial-ankle pulse wave velocity, *BMI* body mass index, *eGFR* estimated glomerular filtration rate, *GGT* γ-glutamyltranspeptidase, *HDL-C* high-density lipoprotein cholesterol, *LDL-C* low-density lipoprotein cholesterol, *SBP* systolic pressure, *DBP* Diastole pressure, *Tg* triglyceride, *TC* total cholesterol, *ABI* ankle-brachial index

### Univariate analysis

The results of univariate analysis are shown in Table 2. The results of univariate analysis showed that age, SBP, DBP, fasting glucose, GGT, uric acid, TC and TG were positively correlated with baPWV. We also found that BMI, HDL-C, LDL, smoking status (current and ex-smoking), ABI, exercise status, alcoholic consumption and AST/ALT were not associated with baPWV, whereas e-GFR and female gender were negatively associated with higher bapWV.

**Table 2:** The results of univariate analysis

baPWV	Statistics	β(95%CI), *P* value
Sex		
male	372 (57.6%)	ref
female	274 (42.4%)	−47.9 (−84.9, −10.8) 0.012
Age	51.5 ± 9.6	13.2 (11.5, 14.8) < 0.001
BMI	22.1 ± 2.4	1.6 (− 5.9, 9.2) 0.669
SBP	117.5 ± 14.6	8.1 (7.0, 9.2) < 0.001
DBP	74.2 ± 9.8	10.4 (8.7, 12.1) < 0.001
γGTP	22.6 ± 22.3	1.3 (0.5, 2.2) 0.001
Fasting glucose	95.2 ± 9.0	6.3 (4.3, 8.3) < 0.001
Uric acid	5.0 ± 1.3	28.3 (14.2, 42.3) < 0.001
TC	206.9 ± 35.2	0.9 (0.4, 1.4) 0.001
TG	84.9 ± 69.3	0.5 (0.3, 0.8) < 0.001
HDL-C	56.7 ± 14.6	−0.4 (−1.6, 0.9) 0.553
LDL	124.1 ± 30.9	0.5 (−0.1, 1.1) 0.083
Current.Smoker		
no	517 (80.0%)	0
yes	129 (20.0%)	−12.6 (−58.6, 33.4) 0.593
Ex-Smoker		
no	346 (53.6%)	0
yes	300 (46.4%)	11.1 (−25.8, 47.9) 0.556
Regular excercise		
no	488 (77.0%)	0
yes	146 (23.0%)	13.6 (−29.3, 56.4) 0.535
e-GFR	70.9 ± 12.1	−5.4 (−6.9, −4.0) < 0.001
ABI	1.2 ± 0.5	37.7 (−0.8, 76.2) 0.056
Alcoholic consumption (g/week)		
< 40	419 (65.4%)	0
> =40, < 140	107 (16.7%)	5.0 (−45.6, 55.7) 0.845
> =140, < 280	57 (8.9%)	20.0 (−46.0, 86.1) 0.552
> =280	58 (9.0%)	48.9 (−16.6, 114.4) 0.144
AST/ALT(per 0.1 change)	11.4 ± 3.6	4.1 (−1.1, 9.2) 0.122

### The relationship between AST/ALT and baPWV

Univariate linear regression models were used to evaluate the associations between AST/ALT and baPWV. Meanwhile, we show the non-adjusted and adjusted models in Table 3. In the crude model, AST/ALT showed no correlation with baPWV (β = 4.1, 95% confidence interval (CI): − 1.1 to 9.2, *P* = 0.122). In the minimally adjusted model (adjusted age, sex), the effect size showed an obvious change (β = 0.9, 95%CI: -3.7 to 5.6, *P* = 0.690). After adjusting for other covariates, we did not detect any connection in a fully adjusted model (β = 2.2, 95%CI: -2.0 to 6.4, *P* = 0.311). For the purpose of sensitivity analysis, we also handled AST/ALT as a categorical variable (quartile) and found the same trend (p for the trend was 0.732).

**Table 3:** Relationship between AST/ALT and baPWV in different models

Variable	Crude model (β, 95%CI, P)	Minimally adjusted model (β, 95%CI, P)	Fully adjusted model (β, 95%CI, P)
AST/ALT(per 0.1 change)	4.1 (− 1.1, 9.2) 0.122	0.9 (−3.7, 5.6) 0.690	2.2 (−2.0, 6.4) 0.311
AST/ALT(quartile)			
Q1	Ref	Ref	Ref
Q2	−20.4(−84.0, 43.2) 0.530	−37.5 (−91.1, 16.1) 0.170	− 26.7 (−73.1, 19.7) 0.260
Q3	− 22.7 (−82.0, 36.7) 0.454	− 38.5 (−90.0, 13.0) 0.143	−22.9 (−67.6, 21.8) 0.316
Q4	−9.9(−69.0, 49.2) 0.742	−33.8(− 86.0, 18.4) 0.205	− 16.2 (− 63.5, 31.1) 0.503
P for trend	0.894	0.352	0.732

Crude model: we did not adjust other covariants

Minimally adjusted model: we adjusted age and sex

Fully adjusted model: we adjusted sex; age; BMI; SBP; DBP; γ-GTP; fasting glucose; uric acid; TC; TG; HDL-C; LDL; smoking status (current and ex-); excise; e-GFR; ABIM; alcoholic consumption

CI: confidence interval; Ref: reference

### The analyses of non-linear relationship

In the present study, we analysed the non-linear relationship between AST/ALT and baPWV because AST/ALT is a continuous variable (Fig. 1). We found that the relationship between AST/ALT and baPWV was non-linear (after adjusting age, sex, BMI, SBP, DBP, GGT, uric acid, fasting glucose, TC, LDL, TG, HDL-C, e-GFR, smoking and exercise statuses, fatty liver, alcohol consumption and ABI). By using a two-piecewise linear regression model, we calculated that the inflection point was 13.1. On the left of the inflection point, the effect size, 95%CI and *P* value were − 4.7, − 12.1 to 2.7 and 0.212, respectively. However, we also observed a positive relationship between AST/ALT and baPWV on the right side of the inflection point (10.9, 3.3 to 18.6, 0.005) (Table 4).

**Table 4:** The results of two-piecewise linear regression model

Inflection point of AST/ALT(Per 0.1 change)	Effect size (β)	95%CI	*P* value
< 13.1	− 4.7	− 12.1 to 2.7	0.212
≥13.1	10.9	3.3 to 18.6	0.005

Effect: baPWV Cause: AST/ALT Adjusted: sex; age; BMI; SBP; DBP; γ-GTP; fasting glucose; uric acid; TC; TG; HDL-C; LDL; smoking status (current and ex-); excise; e-GFR; ABIM; alcoholic consumption

### The results of subgroup analyses

As shown in Table 5, the test for interactions was significant for age (P for interaction = 0.001), while the test for interactions were not statistically significant for alcohol consumption, sex, current smoking, ex-smoking, exercise status, BMI, hypertension and uric acid (*P* values for interactions were larger than 0.05). We observed that there was evidence for an interaction AST/ALT and age. The effect sizes of AST/ALT on baPWV showed significant differences in different generations. AST/ALT was positively associated with baPWV in subjects who were less than 60 years old (β = 15.6, 95%CI (6.3, 25.0)). It was noted that Takuya Fukuda et al. collected the menopausal status in raw data; therefore, we also adjusted this in females. Compared with no-adjusted menopausal status (1.3 (− 4.9, 7.6)), however, the trend of AST/ALT on baPWV (5.4 (− 2.7, 13.5)) was not altered after adjusting for menopausal status.

**Table 5:** Effect size of AST/ALT on baPWV in prespecified and exploratory subgroups

Characteristic	No of participants	Effect size(95%CI)	P for interaction
Age (year)			0.001
≤ 60	98	15.6 (6.3, 25.0)	
> 60	531	−0.8 (−5.7, 4.1)	
Sex			0.32
male	372	5.5 (−0.5, 11.5)	
female	274	1.3 (−4.9, 7.6)	
		5.4 (−2.7, 13.5)*	
Current smoking			0.83
no	517	3.0 (−1.6, 7.5)	
yes	129	1.5 (−11.1, 14.2)	
Ex-Smoking			0.88
no	346	2.6 (−3.2, 8.4)	
yes	300	3.2 (−3.1, 9.5)	
Regular Exercise (> 1 week)			0.08
no	488	0.2 (−4.6, 5.1)	
yes	146	8.8 (0.1, 17.5)	
Alcohol consumption			0.54
≤ 40 (g/week)	419	1.1 (−4.1, 6.3)	
40–140	107	8.1 (−2.8, 18.9)	
140–280	57	6.3 (−14.7, 27.4)	
> 280	58	8.8 (−8.0, 25.7)	
BMI			0.54
< 18.5	36	11.7 (−10.8, 34.1)	
> =18.5, < 23	386	5.8 (0.4, 11.2)	
> =23	224	1.6 (−6.2, 9.5)	
Hypertension			0.90
no	585	3.3 (−1.4, 7.9)	
yes	61	2.2 (−14.3, 18.7)	
Uric acid (tertile)			032
low	298	3.9 (−3.7, 11.5)	
middle	308	−3.8 (−12.9, 5.2)	
high	306	4.5 (−5.4, 14.4)	

Note 1:Above model adjusted for sex; age; BMI; SBP; DBP; γ-GTP; fasting glucose; uric acid; TC; TG; HDL-C; LDL; smoking status (current and ex-); excise; e-GFR; ABIM; alcoholic consumption

Note 2:In each case, the model is not adjusted for the stratification variable

Note 3: * adjusted menopausal status + sex; age; BMI; SBP; DBP; γ-GTP; fasting glucose; uric acid; TC; TG; HDL-C; LDL; smoking status (current and ex-); excise; e-GFR; ABIM; alcoholic consumption

## Discussion

In the present study, we used GLM and GAM models to elucidate the relationship between AST/ALT and baPWV among participants. As is shown in the fully adjusted model, AST/ALT was not associated with baPWV. When we handled AST/ALT as a categorical variable, the same trend was observed. However, the results obtained from GAM and two-piecewise linear regression model showed that the relationship between AST/ALT and baPWV was non-linear, and the correlations between AST/ALT and baPWV were different on the left and right sides of the inflection point (AST/ALT = 13.1). AST/ALT, as assessed at baseline, was not statistically significant on the left side of the inflection point, but AST/ALT was positively associated with baPWV on the right of the inflection point. Interestingly, we also found they have positive correlation with baPWV in participants who were less than 60 years old.

We conducted a PubMed search using the key words ‘brachial-ankle pulse wave velocity’ and ‘AST/ALT’, simultaneously. Three scientific papers were retrieved from the database as of the end of October 2017. All of these studies showed that AST and ALT were associated with baPWV [11]. However, none of them discussed the connection between AST/ALT and baPWV. To our knowledge, this is the first study to investigate the relationship between AST/ALT and baPWV.

The ratio of the serum activities of AST and ALT, also known as the De Ritis ratio, was first described by Fernando De Ritis in 1957. It is commonly used to assess liver function and reflects the severity of liver disease. Previous studies reported that AST/ALT was more helpful for identifying heavy drinking in the NHANES study than when they were used alone [12]. Since then, the value of AST/ALT in cardiovascular and cerebrovascular diseases has gradually attracted the consideration of researchers [13, 14]. Several studies demonstrated that elevated AST/ALT, assessed at baseline, is an independent risk factor for morbidity and mortality in cardiovascular disease [6, 15, 16]. Since baPWV is also associated with cardiovascular morbidity and mortality, the potential clinical value of this study is that AST/ALT may be used for the assessment of arterial stiffness in the future. After all, compared with baPWV, AST/ALT is simpler and more convenient.

Subgroup analysis is extremely important for a scientific study [8]. In the present study, we used age, sex, current smoking, ex-smoking, exercise status, fatty liver, BMI, hypertension, uric acid and alcoholic consumption as stratification variables, of which only age was found. Given that similar results were not reported by previous studies, we could not explain why the linearly increasing trend between AST/ALT and baPWV occurred only in participants younger than 60 years.

Our study has a number of strengths. First, we not only use the generalized linear model to evaluate the linear relationship between AST/ALT and baPWV but also use the generalized additive model to clarify their nonlinear relationship. GAM has obvious advantages in dealing with non-linear relations, can handle non-parametric smoothing and will fit a regression spline to the data. The use of GAM will help us to better discover the real relationships between exposures and outcomes. Second, this study is an observational study, including unavoidable potential confounders; therefore, we used strict statistical adjustment to minimize residual confounding. Third, the effect modifier factor analysis improves the use of the data. The positive association of AST/ALT and baPWV in subjects younger than 60 years was found in the subgroup analysis. Fourth, we had the positive finding that AST/ALT was greater than 1.31 (13.1, per 0.1 change in the text), and for every 0.1 unit increase in AST/ALT, the baPWV increased by 10.9. The clinical value of this finding is that the association of AST/ALT and baPWV can only be observed when AST/ALT reaches a certain threshold.

There are some limitations in our study. First, this study is an analytical cross-sectional study, and thus, it provides only weak evidence of associations between exposure and outcome; it is difficult to distinguish cause and effect. Second, as the study population contains only Japanese participants, it may be not generalizable to other ethnic groups. Third, due to raw data limitations, we cannot adjust the history of atherosclerosis-related diseases since arterial stiffness is strictly associated with atherosclerosis. Besides, correlations between insulin resistance, AST/ALT and arterial stiffness were not performed due to the raw data limitations. Similarly, we could not investigate the plasma levels of inflammatory markers such as tumour necrosis factor, interleukin and high-sensitivity C-reactive protein and their possible correlations with HDL-C ratio and arterial stiffness.

## Conclusion

The relationship between AST/ALT and baPWV is non-linear. AST/ALT is positively correlated with baPWV when AST/ALT (per 0.1 change) is larger than 13.1.

## Abbreviations

ABI: Ankle-brachial index
AST/ALT: aspartate aminotransferase to alanine aminotransferase
baPWV: Brachial-ankle pulse wave velocity
eGFR: Estimated glomerular filtration rate
LDL: Low density lipoprotein
TC: Total cholesterol
